# Reduced synaptic function of Kainate receptors in the insular cortex of Fmr1 Knock-out mice

**DOI:** 10.1186/s13041-018-0396-1

**Published:** 2018-09-21

**Authors:** Shuang Qiu, Yu Wu, Xinyou Lv, Xia Li, Min Zhuo, Kohei Koga

**Affiliations:** 1 Center for Neuron and Disease, Frontier Institute of Science and Technology, Xi’an Jiaotong University, Xi’an, 710049 China; 2 Department of Physiology, Faculty of Medicine, University of Toronto, Medical Science Building, 1 King’s College Circle, Toronto, ON M5S 1A8 Canada; 3 Department of Neurobiology, Key Laboratory of Medical Neurobiology of the Ministry of Health of China, Zhejiang University School of Medicine, Zhejiang, 310058 Hangzhou China; 4 Department of Neurology, The First Affiliated Hospital, Zhejiang University School of Medicine, Zhejiang, 310003 Hangzhou China; 5 Department of Neurophysiology, Hyogo College of Medicine, Nishinomiya, Hyogo 663-8501 Japan

**Keywords:** FMRP, Fragile X syndrome, Insular cortex, Kainate receptor, Internalization, GluK1, GluK2

## Abstract

Fragile X syndrome is caused by the loss of fragile X mental retardation protein (FMRP). Kainate receptor (KAR) is a subfamily of ionotropic glutamate receptors (iGluR) that acts mainly as a neuromodulator of synaptic transmission and neuronal excitability. However, little is known about the changes of synaptic KAR in the cortical area of Fmr1 KO mice. In this study, we performed whole-cell patch-clamp recordings from layer II/III pyramidal neurons in the insular cortex of Fmr1 KO mice. We found that KARs mediated currents were reduced in Fmr1 KO mice. KARs were mainly located in the synaptosomal fraction of the insular cortex. The abundance of KAR subunit GluK1 and GluK2/3 in the synaptosome was reduced in Fmr1 KO mice, whereas the total expressions of these KARs subunits were not changed. Finally, lack of FMRP impairs subsequent internalization of surface GluK2 after KAR activation, while having no effect on the surface GluK2 expression. Our studies provide evidence indicating that loss of FMRP leads to the abnormal function and localization of KARs. This finding implies a new molecular mechanism for Fragile X syndrome.

## Introduction

Fragile X syndrome (FXS) is the most common monogenic cause of autism and inherited mental impairment [1, 2]. The FXS is almost exclusively caused by an expansion of a trinucleotide repeat (CGG) repeat in the 5′ untranslated region of the X-linked fragile X mental retardation 1 (*Fmr1*) gene. Mutation in the Fmr1 gene leads to a failure to express the fragile X mental retardation protein (FMRP), which functions in repressing local translation at the synapse and regulating mRNA trafficking and stability [3–7]. Fmr1 knockout (KO) mice are sufficient to generate a mouse model for FXS and exhibit cognitive deficits and abnormal plasticity in the cortex or hippocampus.

Local protein synthesis is required for long-term synaptic plasticity that stores memories and is orchestrated by the action of glutamate receptors. Activation of metabotropic glutamate receptors (mGluR) produces long-term depression (LTD), which involves local protein synthesis and degradation [8]. In Fmr1 KO mice, mGluR-dependent LTD is strongly increased, mainly due to deregulation of local protein synthesis, and the exaggerated mGluR signaling contributes to many of the synaptic phenotypes in FXS [9, 10].

Besides mGluR, ionotropic glutamate receptors (iGluRs), including N-methyl-D-aspartate receptor (NMDAR) and α-amino-3-hydroxyl-S-methylisoazole-4-propionate receptor (AMPAR), have been identified to be involved in FMRP. FMRP is critical for NMDAR-dependent LTP in the cingulate cortex (ACC) and prefrontal cortex (PFC) [11, 12]. Spike timing-dependent plasticity that requires NMDAR activation is attenuated in neocortical slices from early postnatal Fmr1 KO mice [13]. Moreover, Fmr1 KO mice show impaired performance in an NMDAR-dependent context discrimination task [14]. FMRP is involved in regulating the internalization of AMPARs [15, 16]. Surface expression and phosphorylation of AMPAR subunit GluA1 in response to dopaminergic D_1_ receptor stimulation are reduced in PFC neurons from Fmr1 KO mice [17].

Kainate receptor (KAR) is another subtype of iGluRs, which is present at both presynaptic and postsynaptic sites in the cortex. Both KAR GluK1 and GluK2 subunits involved in KARs mediated transmission in pyramidal neurons from surface layers (layer II/III) of the adult mice ACC and insular cortex [18–20]. Although KAR mediated currents are much smaller in the ACC and insular cortex compared with AMPAR mediated currents, KAR participates in various physiological functions, behavors and pathological conditions by transgenic mice or pharmacological inhibitors of GluK receptors [18, 21–23]. Recently, it was reported that FMRP plays an important role in GluK1 containing GluKRs dependent pre-LTP in ACC neurons [24]. However, it is still unclear whether the function and expression of KARs is altered in Fmr1 KO mice.

In the present study, we investigate the function and expression of KAR in the insular cortex using Fmr1 KO mice. In vitro whole-cell patch-clamp recordings from layer II/III pyramidal neurons showed that Fmr1 KO mice reduced KARs mediated functions in the insular cortex. KAR was enriched in the synaptosome in Fmr1 WT mice, while the abundance of KAR in the synaptosome was decreased in Fmr1 KO mice. Additionally, Kainate-induced internalization of surface GluK2 was impaired in Fmr1 KO mice. These findings provide evidence that FMRP participates in regulating KAR localization and trafficking in the insular cortex.

## Methods

### Animals

Adult male Fmr1 wild-type (WT) and Fmr1 knock-out (KO) mice (8 to 12 weeks of age) of the FVB. 129P2-Fmr1tm1Cgr strain used in the experiment were obtained from Dr. WT Greenough (University of Illinois, Champaign, IL). Mice were housed under a 12-h light and dark cycle with food and water provided ad libitum. This study was carried out in accordance with the principles of the Basel Declaration and recommendations followed by the guidelines set from The Canadian Council on Animal Care. In addition, the animal experiments were performed in accordance with the ethical guidelines of the Zhejiang University Animal Experimentation Committee and were in complete compliance with the National Institutes of Health Guide for the Care and Use of Laboratory Animals. All of the protocols were approved by The Animal Care and Use Committee of University of Toronto, Xi’an Jiaotong University and Zhejiang University.

### Whole-cell patch-clamp recordings from insular cortex slices

Mice were anesthetized by isoflurane (1–2%). We prepared transverse brain slices of the insular cortex (300 μm) by standard methods [18–20]. Slices were kept in a room temperature submerged recovery chamber with an oxygenated (95% O_2_–5% CO_2_) solution containing (in mM) 124 NaCl, 25 NaHCO_3_, 2.5 KCl, 1 KH_2_PO_4_, 2 CaCl_2_, 2 MgSO_4_, and 10 glucose. After a one hr. recovery period, brain slices were brought into a recording chamber on the stage of an Axioskop 2FS microscope (Carl Zeiss) equipped with infrared differential interference contrast optics for whole-cell patch-clamp recordings. Excitatory postsynaptic currents (EPSCs) were obtained from pyramidal neurons in layer II/III with an Axon 200B amplifier (Axon Instruments) in the insular cortex, and electrical stimulation was given by a bipolar tungsten electrode placed in layer *V*/VI of the insular cortex [19]. The evoked stimulations were given every 30 s as control test pulses. Repetitive high frequency stimulations were delivered at 200 Hz (5, 10, or 20 shocks) for frequency facilitations. Under the voltage-clamp mode, recording electrodes (2–5 MΩ) with the pipette solution composed of (in mM) 120 Cs-gluconate, 5 NaCl, 1 MgCl_2_ 0.5 EGTA, 2 Mg-ATP, 0.1 Na_3_GTP, 10 HEPES, and 2 lidocanine N-methyl bromide quaternary salt (QX-314), pH 7.2, 280–300 mosmol/l [18, 19]. The initial access resistance was between 15 and 30 MΩ, and was recorded during the experiment. Data were stopped when the access resistance became more that 15% during the experiment. We used the filter at 1 kHz and the data was digitized at 10 kHz. The holding membrane potential was at − 60 mV during the experiments. All experiments were done in the presence of picrotoxin (PTX; 100 μM) and D-2-amino- 5-phosphonopentanoic acid (AP-5; 50 μM). We recorded time constants of EPSCs by fitting one exponential function to the falling phase of the currents.

### Drugs and antibodies

Kainate (KA) was purchased from Tocris Cookson (Bristol, UK), Picrotoxin (PTX), D-2-amino-5-phosphono-pentanoic acid (AP-5), 1-(4-aminophenyl)-4-methyl-7,8-methylenedioxy- 5H-2,3-benzodiazepine (GYKI53655), 6-cyano-7-nitroquinoxalene-2,3-dione (CNQX), phosphatase inhibitor cocktail 2 and 3 were obtained from Sigma-Aldrich (St. Louis, MO). Antibodies were used: anti-PSD95 (#3450, 1:1000) were from Cell Signaling Technology, anti-synaptophysin (S55768, 1:5000) and anti-β-actin (A2066, 1:1000) were purchased from Sigma, anti-rab5 (sc-46692, 1:500) were from Santa Cruz Biotechnology; anti-GluN2A (AB1555, 1:2000), anti-GluN2B (AB1557, 1:500), anti-GluK1 (07–258, 1:500) and anti-GluK2/3 (04–921, 1:1000) were purchased from Millipore Bioscience Research Reagents.

### Primary culture of the cortical neurons

Cortical neuronal cultures from Fmr1 WT or Fmr1 KO mice were prepared by a previously described protocol [25, 26]. Cortical neurons from embryonic day 17 (E17) rats were cultured as described previously [27]. Cultured neurons were incubated in 5% CO_2_ humidified incubator at 37 °C and used for experiments at 14 days in vitro (DIV 14).

### Brain slices preparations

Brain slices were prepared as previously described [25]. The anatomical terminology was based on the Atlas of Franklin and Paxinos [28]. Fmr1 WT or Fmr1 KO mice were anesthetized with halothane (2%) and 300 μm thickness of brain slices containing the insular cortex were cut with a vibratome in oxygenated artificial cerebrospinal fluid (ACSF) containing 124 mM NaCl, 2 mM KCl, 26 mM NaHCO_3_, 2 mM CaCl_2_, 2 mM MgSO_4_, 1 mM NaH_2_PO_4_, and 10 mM D-glucose (pH 7.4) at 4 °C. For performing electrophysiological analysis, the brain slices were put in a submerged recover chamber with oxygenated ACSF at room temperature.

### Subcellular fractionation

Subcellular fractionation was conducted by a previous protocol [25, 26]. The insular cortex was dissected in cold ACSF and homogenized in 0.32 M sucrose buffer (10 mM sucrose, 10 mM Hepes, pH 7.4) containing a protease inhibitor cocktail. Samples were centrifuged (1000 g, 10 min, 4 °C) to yield the nuclear enriched pellet and the S1 fraction. The S1 fraction was then centrifuged (12,000 g, 20 min, 4 °C) to obtain the supernant (S2) and pellet (P2; crude synaptosomal membranes) fraction. The P2 pellet was then re-suspended in radioimmunoprecipitation assay (RIPA) buffer [50 mM tris-Cl (pH 7.6), 150 mM NaCl, 1 mM EDTA, 0.1% SDS, 1% NP-40, 0.5% sodium deoxycholate, 1 mM dithiothreitol]. To further digest synaptosomes, we re-suspended the P2 pellet in 4 mM Hepes buffer (4 mM Hepes, 1 mM EDTA, pH 7.4) and then centrifuged (12,000 g, 20 min, 4 °C). Re-suspension and centrifugation were repeated. The resulting pellet was resuspended in buffer A (20 mM Hepes, 0.5% Triton X-100, 100 mM NaCl, pH 7.2) and rotated slowly (15 min, 4 °C), followed by centrifugation (12,000 g, 20 min, 4 °C). The supernatant (Triton X-100 soluble non-PSD fraction) was retained. The pellet was resuspended in buffer B (20 mM Hepes, 0.15 mM NaCl, 1% deoxycholic acid, 1% Triton X-100, 1% SDS, 1 mM dithiothreitol, pH 7.5), followed by gentle rotating (1 h, 4 °C) and centrifugation (10,000 g, 15 min, 4 °C). The supernatant (Triton X-100 insoluble PSD fraction) was retained and the pellet was discarded.

### Surface Biotinylation assay of KARs

Surface biotinylation assay was conducted with cultured cortical neurons following a previous protocol [29, 30]. We used primary cultured cortical neurons at DIV 14 treated with or without Kainate for indicated times. The neurons were washed twice with ice-cold PBS containing 1 mM MgCl_2_ and 0.1 mM CaCl_2_ (PBS+) and incubated with 1 mg/ml EZ-Link Sulfo-NHS-SS-biotin (Thermo Fisher Scientific) in PBS+ for 30 min at 4 °C with gentle constant shake. Subsequently, the cells were washed with an ice-cold quenching buffer (50 mM glycine in PBS+) and lysed in lysis buffer (1% triton X-100, 0.5% sodium deoxycholate in PBS) at 4 °C for 30 min. Followed by 15 min centrifugation at 4 °C, the supernatant was incubated with streptavidin-Sepharose beads (Thermo Fisher Scientific) overnight at 4 °C. The bound proteins were eluted by a 1.5 × SDS buffer and detected by western blot.

### Western blot analysis

Western blot was performed as described previously [25, 31]. Protein concentrations were normalized with the Bradford assay. Equal amounts of protein extract were loaded on SDS-PAGE gels, and separated proteins were transferred onto polyvinylidene membranes at 4 °C. The membranes were blocked with a blocking buffer [5% milk in TBST (tris-buffered saline with Tween 20)] and incubated with primary antibodies (4 °C, overnight). After being washed three times, the membranes were incubated with an appropriate HRP-coupled secondary antibody for another 1 h, and then detected with the Western Lightning Chemiluminescence Reagent Plus. The density of the immunoblots was analyzed with ImageJ.

### Data analysis

The data are shown as means ± SEM. Statistical analysis of differences between two groups was conducted by unpaired, two-tailed Student’s t-test or Mann-Whitney rank sum test, based on a normality test (Shapiro-Wilk) of the data. We performed a two-way ANOVA and Tukey test for post hoc test when there were 2 independent variables. A probability value of *P* < 0.05 was considered as significant.

## Results

### Subcellular distribution of KARs in the insular cortex

**Fig. 1:**
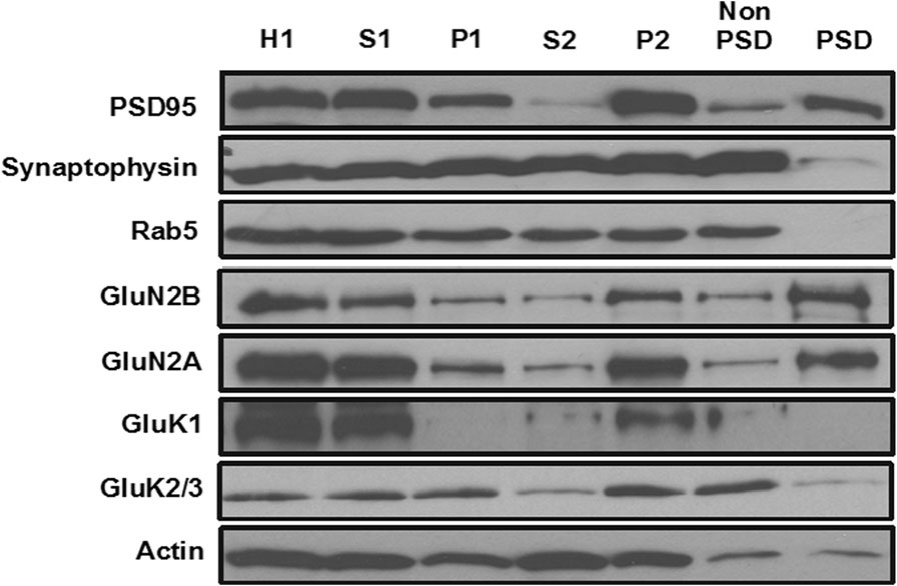
Distribution of KARs in the insular cortex from Fmr1 WT mice. Presynaptic marker synaptophysin, postsynaptic marker PSD95, NMDAR subunits GluN2B and GluN2A, KAR subunits GluK1 and GluK2/3 or synaptic protein Rab5 were analyzed by Western blot in the homogenates (H1, 10 μg), postnuclear supernatant (S1, 5 μg), nuclei and large debris pellet (P1, 10 μg), cytosomes (S2, 5 μg), crude synaptosomal membrane (P2, 5 μg), non-PSD (5 μg) or PSD (10 μg) fractions of the insular cortex in Fmr1 WT mice. This experiment was repeated three times

We examined the subcellular distribution of KARs in the insular cortex of Fmr1 WT mice by synaptosome fractionation. Crude synaptosomal fraction (P2) contains pre- and postsynaptic structures [32]. Further digestion of synaptosome yields a Triton X-100 insoluble PSD fraction and a Triton X-100 soluble non-PSD fraction [25, 33]. As shown in Fig. 1, PSD95 was enriched in the PSD fraction, while Synaptophysin and Rab5 were in the non-PSD fraction (Fig. 1). NMDAR subunits GluN1 and GluN2A were mainly located in the PSD fraction, which is inconsistent with previous findings [25]. GluK1 and GluK2/3 were highly enriched in the P2 fraction (synaptosomal pellet). Unlike NMDARs, both GluK1 and GluK2/3 were mainly distributed in the non-PSD fraction, but not in the PSD fraction (Fig. 1).

### Fmr1 KO mice reduced KARs mediated EPSCs in pyramidal neurons from layer II/III of the insular cortex

**Fig. 2:**
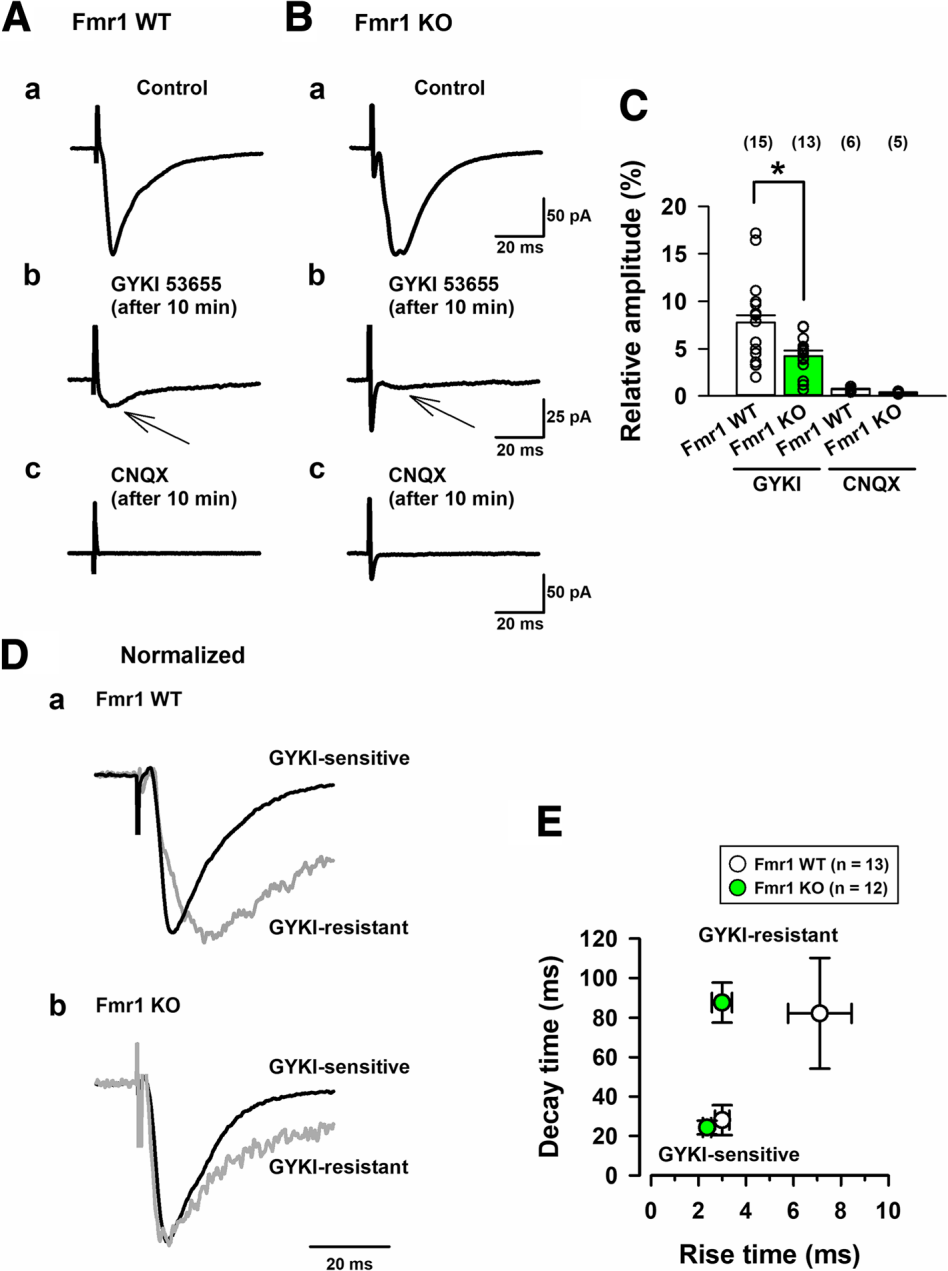
Fmr1 KO mice reduced KARs-mediated EPSCs in pyramidal neurons of layer II/III insular cortex. **a**, **b**, To detect KARs-mediated EPSCs, AMPAR/KAR mediated EPSCs were obtained in the presence of GABA_A_ receptor antagonist, PTX (100 μM) and a NMDARs angatonist, AP-5 (50 μM) for 5 min in Fmr1 WT (Aa) and KO mice (Ba). 10 min after the perfusion of an AMPAR antagonist, GYKI 53655 (100 μM), a residual current remained (A-Bb). The small currents could be totally blocked by a AMPAR/KARs antagonist, CNQX (20 μM) in Fmr1 WT (Ac) and Fmr1 KO mice (Bc). **c**, Statistical results of the percentage of EPSCs in the presence of GYKI 53655 (*n* = 15 in 11 Fmr1 WT mice, *n* = 13 in 9 Fmr1 KO mice), and CNQX (*n* = 6 in 6 Fmr1 WT mice, *n* = 5 in 4 Fmr1 KO mice). Compared with KARs mediated currents in Fmr1 WT mice, KAR-mediated currents in Fmr1 KO mice were significantly decreased (*P < 0.05). **d**, KA receptor-mediated EPSCs show slower kinetics in Fmr1 WT and Fmr1 KO mice. Normalized traces of GYKI53655-sensitive and GYKI53655-resistant EPSCs were recorded. **e**, The data of averaged rise time and decay time in GYKI-sensitive and GYKI-resistant traces. The rise time in GYKI53655-resistant EPSCs between Fmr1 WT and Fmr1 KO mice were significantly different (n = 13 in 10 Fmr1 WT mice, *n* = 12 in 10 Fmr1 KO mice) (*P < 0.05)

We tested whether Fmr1 KO mice could alter KARs mediated glutamatergic transmissions in insular cortex neurons. We conducted in vitro whole-cell patch-clamp recordings to visually identify pyramidal cells in layers II/III of the insular cortex slices from adult Fmr1 WT and Fmr1 KO mice [19]. To induce evoked KARs mediated currents, a stimulation electrode was located in layer *V*/VI of the insular cortex. In the presence of the GABA_A_ receptor antagonist, PTX (100 μM) and a selective NMDA receptor antagonist, AP-5 (50 μM), single-pulse stimulation evoked EPSCs could be recorded in Fmr1 WT mice (Fig. 2A). After a stable EPSCs baseline of at least 5 min was observed, we applied the potent AMPA receptor antagonist, GYKI53655 (100 μM) in the bath solution. A small residual EPSCs lasted in the presence of GYKI 53655 10 min after the application. The residual EPSCs were considered as KAR mediated currents. Lastly, the AMPAR/KAR antagonist, CNQX (20 μM) was applied, and the bath application of CNQX completely abolished the small GYKI 53655-resistant current. These results suggest that the currents were mediated by KARs (Fig. 2A).

Next, we recorded KAR mediated currents in Fmr1 KO mice. The insular cortex neurons in Fmr1 KO mice showed small KAR mediated currents (Fig. 2B). The avaraged data of KA mediated EPSCs are shown in Fig. 2C. The averaged amplitudes of KARs EPSCs in Fmr1 WT mice were 7.8 ± 1.2% of the AMPARs/KARs EPSCs as a baseline (averaged AMPA/KA EPSCs: 131.0 ± 5.7 pA; averaged KA EPSCs: 9.8 ± 1.4 pA, *n* = 15 neurons in 11 Fmr1 WT mice). These KAR mediated currents in Fmr1 WT mice are similar to the KA currents in C57BL/6 J mice [18, 19]. KAR EPSCs in Fmr1 KO mice were decreased compared with Fmr1 WT mice (AMPA/KA EPSCs: 125.2 ± 16.7 pA; KA EPSCs: 6.1 ± 1.0 pA, *n* = 13 neurons in 9 Fmr1 KO mice, *P < 0.05 vs Fmr1 WT mice). These results suggest that FMRP is critical for KAR mediated synaptic transmission in the insular cortex.

### Fmr1 KO mice show altered kinetics in KAR EPSCs in insular cortex

As shown in Fig. 1D-E, GYKI53655-sensitive and -resistant currents were normalized in Fmr1 WT and Fmr1 KO mice (Fig. 2D). The property of GYKI53655-sensitive currents did not change between the two groups (Fig. 2E). KAR EPSCs (GYKI53655-resistant) displayed slower decay time than AMPA EPSCs (GYKI53655-sensitive) in both groups (82.2 ± 28.0 ms in 13 Fmr1 WT mice, 87.6 ± 10.1 ms in 12 Fmr1 KO mice). However, the rise time (10–90%) of KA EPSCs in Fmr1 KO mice was significantly faster than the time of KAR EPSCs in Fmr1 WT mice (7.1 ± 1.3 ms in Fmr1 WT mice, 3.0 ± 0.4 ms in Fmr1 KO mice, *P < 0.05) (Fig. 2E). Since the slow kinetics in insular cortex neurons of Fmr1 WT mice are similar to those in C57BL/6 J mice [19], a lack of FMRP may alter the functions and kinetics of KARs mediated synaptic transmission in the insular cortex.

### Fmr1 KO mice reduced summation properties of KAR-EPSCs during repetitive high frequency stimulations

**Fig. 3:**
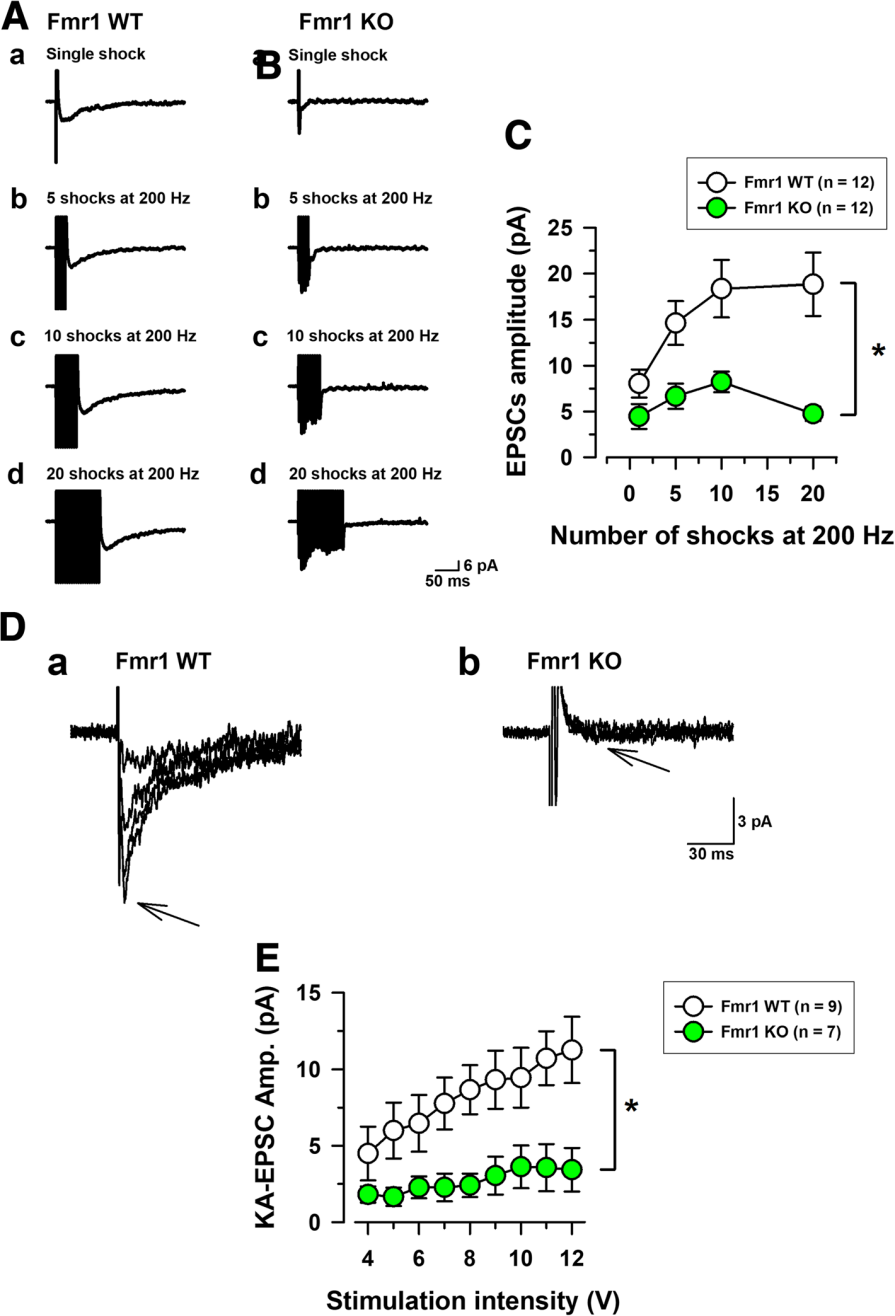
Fmr1 KO mice decreased high-frequency stimulation-dependent summations of KAR mediated EPSCs in insular cortex neurons. **a**, **b**, Representative traces of KA EPSCs recorded by different numbers of stimulations (1, 5, 10, and 20 shocks; a-d, respectively) at the frequency of 200 Hz in Fmr1 WT (*A*) and Fmr1 KO mice (*B*). **c,** Insular cortex neurons in Fmr1 KO mice show strong reductions by these repetitive stimulations (n = 12 in 7 Fmr1 WT mice, n = 12 in 8 Fmr1 WT mice, *P < 0.05). **d**, KARs mediated input-output relationships for single shock-induced KAR-EPSCs showing intensity dependent summation in Fmr1 WT (*A*) and Fmr1 KO mice (*B*). **e**, Neurons in Fmr1 WT mice showed increased amplitudes of KAR-EPSCs that were intensity dependent (*n* = 9 in 7 Fmr1 WT mice), but neurons in Fmr1 KO mice showed no such significant increase (*n* = 7 in 5 Fmr1 KO mice, *P < 0.05)

In most synapses in the brain, repetitive stimulations increase KARs mediated EPSCs [18, 19, 34–36]. Thus, we determined that Fmr1 KO mice could alter the summational properties of KARs mediated synaptic transmissions in the insular cortex (Fig. 3A-C). The repetitive high frequency stimulations at 200 Hz were applied by single, 5, 10 and 20 shocks in Fmr1 WT mice and Fmr1 KO mice (Fig. 3A-B). Figure 3A shows that in the presence of GYKI 53655,small residual KAR EPSCs in Fmr1 WT mice significantly increased in amplitude after repetitive stimulation (8.0 ± 1.5 pA by single stimulation, 14.6 ± 2.4 pA by 5 shocks, 18.4 ± 3.1 pA by 10 shocks and 18.9 ± 3.4 pA by 20 shocks, *n* = 12 in 7 Fmr1 WT mice, Fig. 3C). However, in Fmr1 KO mice, KARs mediated currents induced by 5, 10 and 20 shocks at a 200 Hz train were significantly reduced compared with those in Fmr1 WT mice (4.5 ± 1.4 pA by single stimulation, 6.7 ± 1.4 pA by 5 shocks, 8.2 ± 1.1 pA by 10 shocks and 5.0 ± 0.8 pA by 20 shocks n = 12 in 8 Fmr1 KO mice, *P < 0.05, Fig. 3C).

In order to further verify these results, we recorded the input (stimulation intensity)-output (KA-EPSC amplitude) relationship of KA EPSCs in Fmr1 WT and Fmr1 KO mice (Fig. 3D, E). Fig. 3D showed that Fmr1 WT mice increased the amplitudes of KARs EPSCs by giving stronger stimulation intensity from 4 to 12 V (*n* = 9 neurons in 7 Fmr1 WT, Fig. 3 Da). However, Fmr1 KO mice did not show the increased KAR EPSCs (*n* = 7 neurons in 5 Fmr1 KO, Fig. 3Db). Compared with Fmr1 WT mice, Fmr1 KO mice reduced stimulation intensity-dependent amplitudes of KAR EPSCs (*P < 0.05, Fig. 3E). Taken together, these results indicate that FMRP may play important roles in KAR mediated synaptic transmission within the insular cortex.

### KARs in the synaptosome is reduced in Fmr1 KO mice

**Fig. 4:**
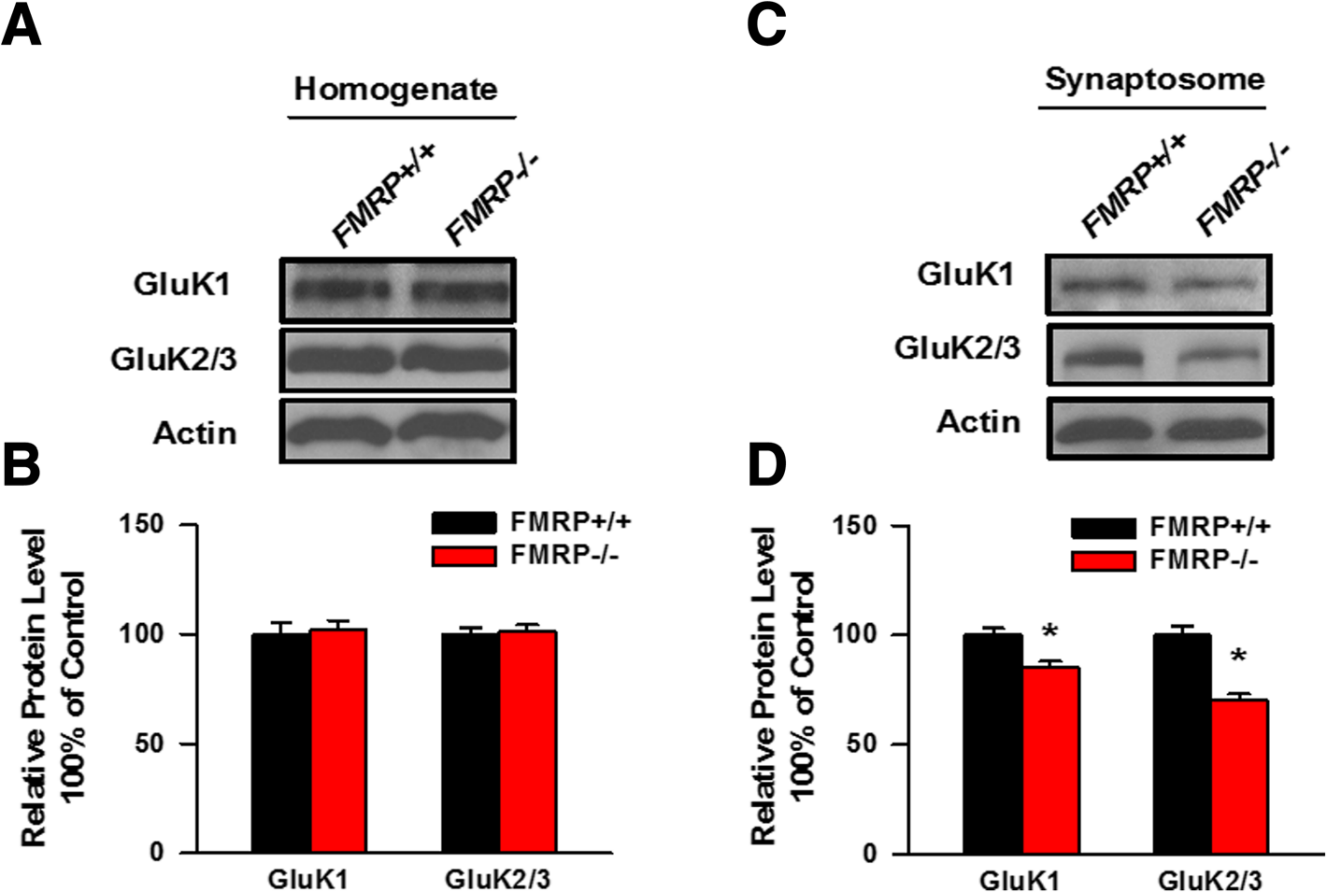
The abundance of KARs in the synaptosome is decreased in Fmr1 KO mice. **a**, **b**, Total expression levels of GluK1 and GluK2/3 in the homogenates fraction of the insular cortex conducted from Fmr1 WT and Fmr1 KO mice were detected by Western blot. The expression levels of GluK1 and GluK2/3 in the homogenates were not altered between Fmr1 WT and Fmr1 KO mice (*n* = 3 mice for each group). **c**, **d**, Expression levels of GluK1 and GluK2/3 in the synaptosome of the insular cortex obtained from Fmr1 WT and Fmr1 KO mice were detected by western blot anaylsis. The expression levels of GluK1 and GluK2/3 in the homogenates was significantly reduced in Fmr1 KO mice compare to Fmr1 WT mice (*n* = 5 mice for each group). *P < 0.05

To further investigate whether the expression level of total KAR subunits or the distribution of KAR subunits in the synaptosome is changed in the insular cortex from Fmr1 WT and Fmr1 KO mice, we performed a biochemical assay to analyze the amount of GluK1 and GluK2/3 in the homogenate or in the synaptosomal fraction of the insular cortex. As shown in Fig. 4A and B, the amount of GluK1 and GluK2/3 was unaltered in the insular homogenate of Fmr1 KO mice compared to Fmr1 WT mice. However, the abundance of GluK1 and GluK2/3 in the synaptosome of the insular cortex was significantly reduced in Fmr1 KO mice compared to Fmr1 WT mice (Fig. 4C and D). These results indicated that deficiency of FMRP results in the reduced synaptic localization of KAR subunits in the insular cortex.

### Evoked KAR currents were unchanged in the cultured neurons from Fmr1 KO mice compared with Fmr1 WT mice

**Fig. 5:**
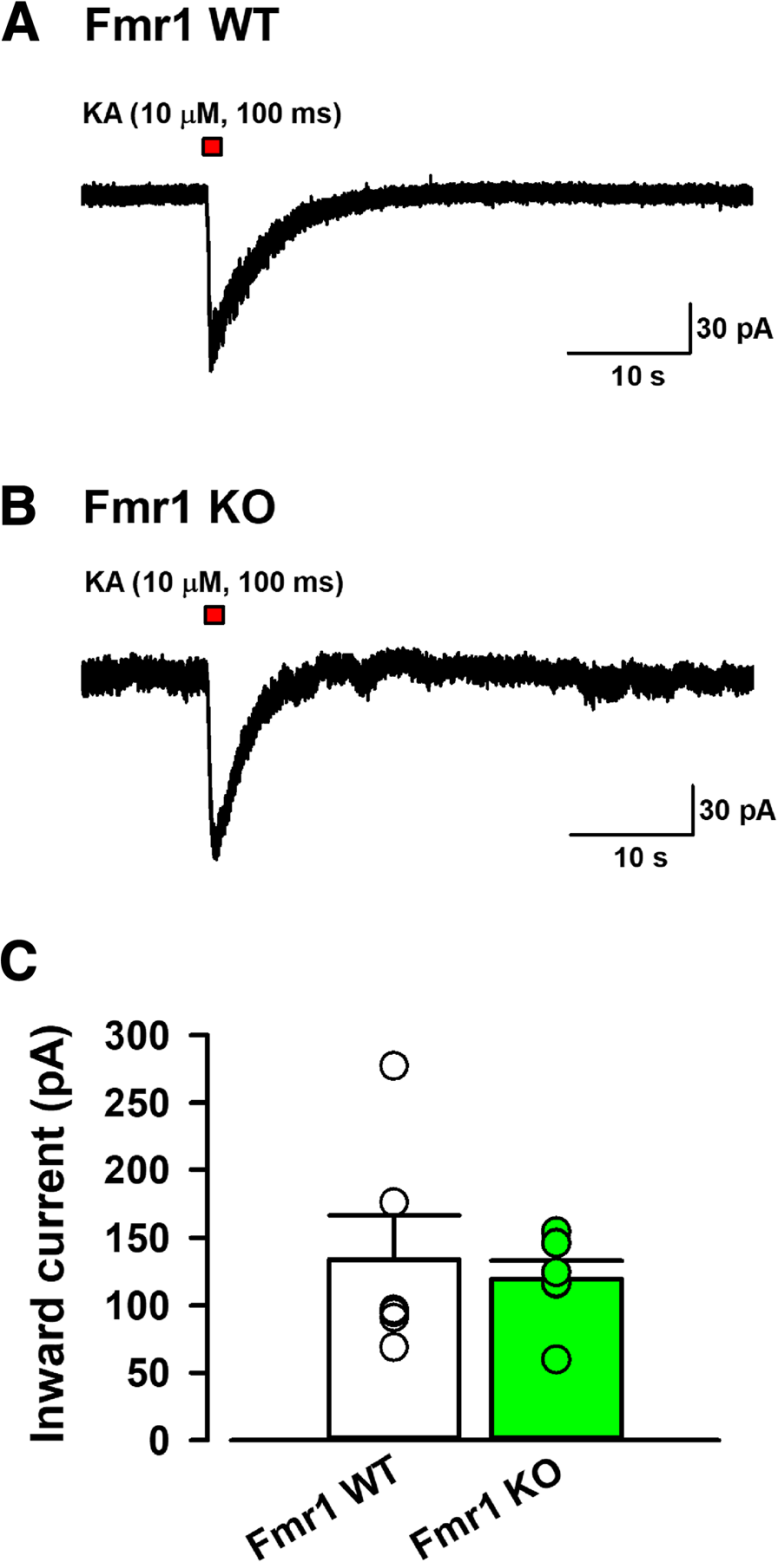
The function of KA receptors in cultured insular cortex neurons from Fmr1 WT and Fmr1 KO mice. **a**, **b**, Typical traces of the KA produced inward current caused by puff perfusion of KA (10 μM) for 100 ms at 15 psi pressure with PTX (100 μM), AP-5 (50 μM) and GYKI53655 (100 μM) in cultured insular cortex neurons from Fmr1 WT (*A*) and Fmr1 KO mice (*B*). **c**, Avaraged data showing that there was no significant difference in KA-induced inward currents between cultured neurons from Fmr1 WT and Fmr1 KO mice (*n* = 6 in 6 Fmr1 WT, n = 6 in 5 Fmr1 KO mice)

To further study the expression and function of KARs, we tested the Kainate-evoked KAR currents in the cultured cortical neurons from Fmr1 WT and Fmr1 KO mice. Kainate (10 μM) was puff-applied (15 psi, 100 ms) in PTX, AP-5 and GYKI 53655 containing ACSF was voltage-clamped under − 60 mV (Fig. 5). The puff application of Kainate produced inward currents in Fmr1 WT and Fmr1 KO mice (Fig. 5A, B). Comparing the KA-mediated currents in cultured neurons from Fmr1 KO mice with those in Fmr1 WT mice, KA-evoked currents were unchanged between Fmr1 KO and Fmr1 WT mice (134.1 ± 32.3 pA, *n* = 6 in 5 Fmr1 WT mice, 119.5 ± 13.6 pA, n = 6 in 6 Fmr1 KO mice, *P* > 0.05, Fig. 5C). These results suggest that the lack of the *Fmr1* gene does not affect the abundance of surface KAR in the cultured cortical neurons.

### Total and surface expression of KARs did not change in the cultured cortical neurons from Fmr1 KO mice or Fmr1 WT mice

**Fig. 6:**
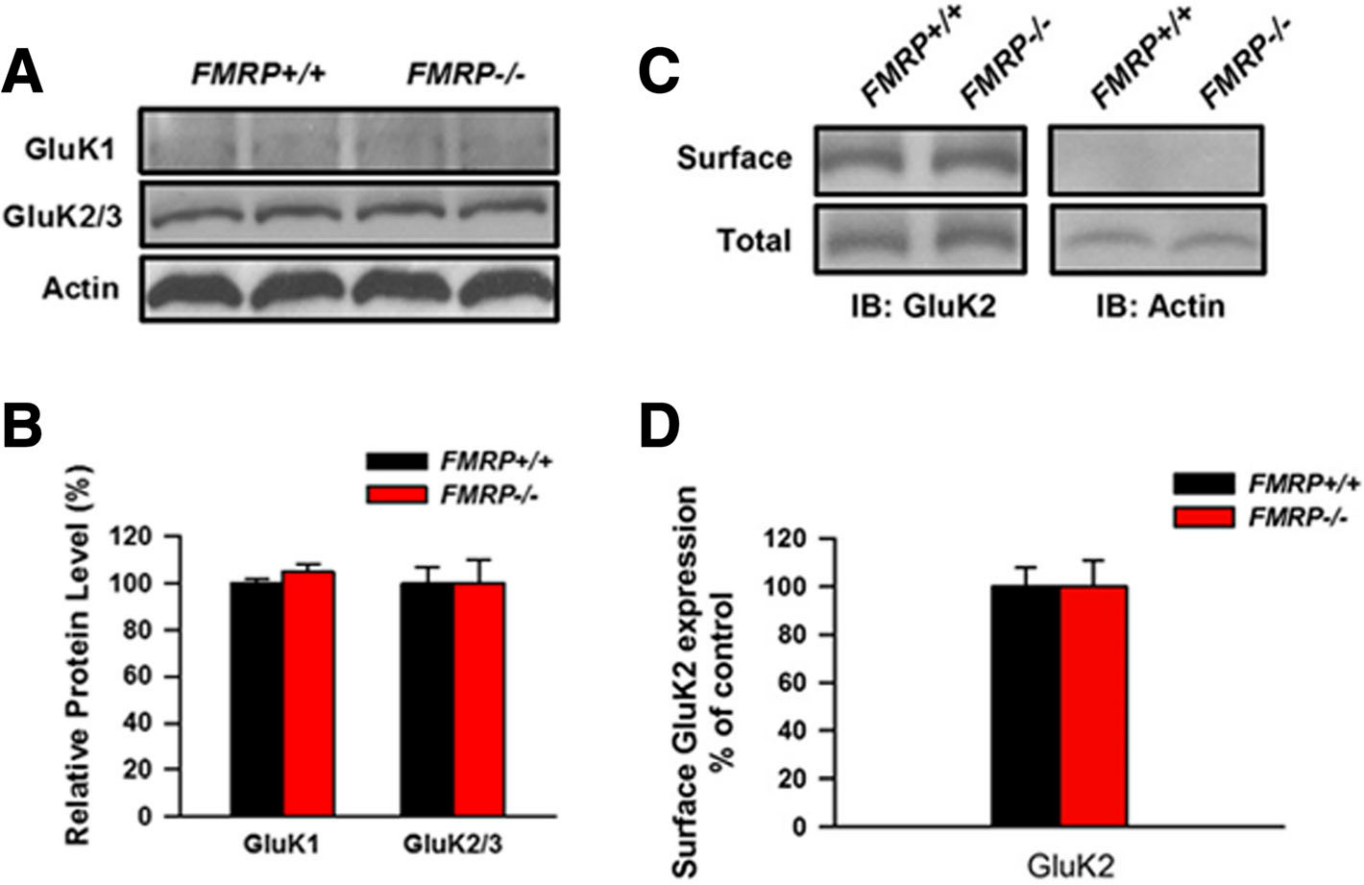
The expression of KARs in the cultured cortical neurons from Fmr1 WT and Fmr1 KO mice. **a**, **b**, The abundance of GluK1 or GluK2/3 in the homogenate of the cultured cortical neurons from Fmr1 WT mice and Fmr1 KO mice showed no change. **c**, **d**, Surface expression levels of GluK2/3 in insular cortex neurons obtained from Fmr1 WT and Fmr1 KO mice were detected by western blot analysis. The surface expression levels of GluK2/3 were not altered between Fmr1 WT and Fmr1 KO mice (n = 3 independent experiments). Actin was used as negative control for surface biotinylation

Next, we cultured cortical neurons from Fmr1 WT and Fmr1 KO mice, and compared the total and surface expression of KARs. As shown in Fig. 6A, the total levels of GluK1 and GluK2/3 showed no significant difference between two groups. We also tested the surface expression level of GluK2/3 in the cultured cortical neurons using surface biotinylation. As shown in Fig. 6B, no difference has been observed between the two groups. These results indicate that FMRP deficiency has no effect on the total expression or surface localization of KARs.

### Activity induced endocytosis of KARs was impaired in cultured cortical neurons from Fmr1 KO mice

**Fig. 7:**
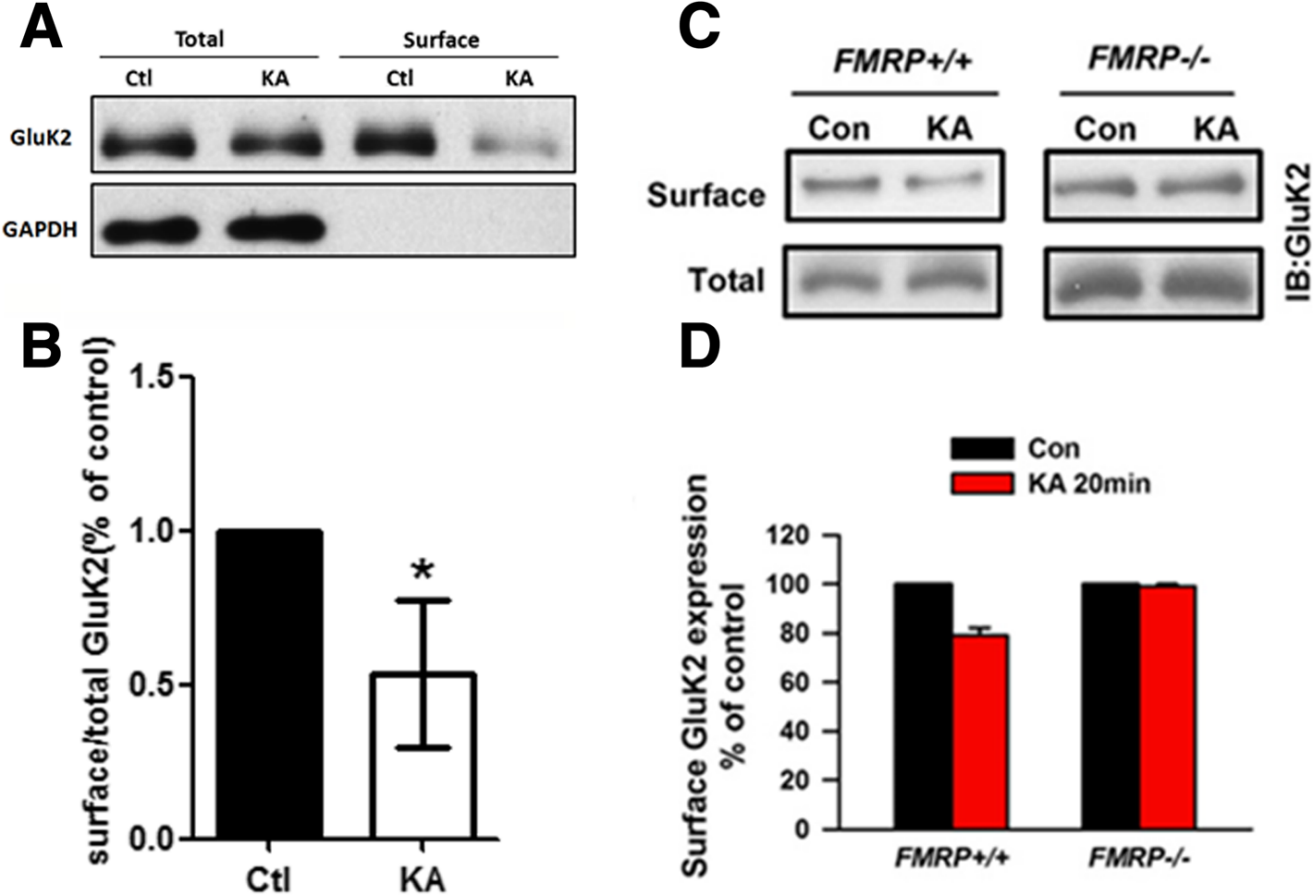
Activity-induced internalization of GluK2 subunit was impaired in cultured cortical neurons from Fmr1 KO mice**. a**, **b**, GluK2/3 subunit undergoes activity induced internalization in the cultured rat cortical neurons (n = 5 independent experiments). *P < 0.05. **c**, **d**, The cultured cortical neurons obtained from Fmr1 WT or Fmr1 KO mice were activated by KA (10 μM) for 20 min and the surface expression of GluK2 was detected by Western blot. The abundance of surface GluK2 in Fmr1 WT mice was significantly decreased upon KA stimulation, whereas that of GluK2 in Fmr1 KO mice did not change (*n* = 4 independent experiments). *P < 0.05

Previous studies indicate that surface localization of KARs is regulated by neuronal activity. For example, stimulation of neurons with kainate acid induced internalization of GluK2 subunits of KARs in the cultured neurons. To test the role of FMRP in KA receptor trafficking, we first set up a biotinylation assay to test the surface-localized KARs in the cultured cortical neurons obtained from the wild-type rat (Fig. 7A,B). We found that the amount of surface GluK2 was significantly decreased when the neurons were stimulated with Kainate acid. Next, we tested the effects of FMRP deficiency on activity-induced KAR trafficking (Fig. 7C,D). In the cortical neurons cultured from Fmr1 WT mice, stimulation with kainate acid induced the internalization of GluK2/3 subunits. However, KA-induced internalization of GluK2/3 subunits was completely blocked in the neurons cultured from Fmr1 KO mice. These results indicate that lack of FMRP impairs activity-induced trafficking of KARs.

## Discussion

The mGluR theory of FXS is well investigated. However, in addition to mGluR, iGluRs are also regulated by FMRP. FMRP is critical for the surface expression and phosphorylation of AMPARs GluA1 subunit in response to dopaminergic D_1_ receptor activation [17]. Furthermore, FMRP is important for NMDA receptor-dependent LTP in the ACC and the hippocampal DG region [11, 14]. Here, we demonstrate that synaptic localization and function of KARs are affected in the insular cortex of Fmr1 KO mice, indicating that KARs may also participate in the pathology of the FXS.

In spite of their slow and small currents, KARs play important roles in cortical neurons. Facilitations of GluK1-containing KARs in the ACC and the insular cortex can regulate glutamatergic and GABAergic transmission [19, 37]. The cortical pre-LTP requires activations of KAR [38]. FMRP signaling also involves the KARs dependent cortical pre-LTP [24]. Importantly, Fmr1 KO mice inhibit the expression of the cingulate pre-LTP. One possible reason for the inhibition of pre-LTP is that Fmr1 KO mice alter catalytic and regulatory parts of PKA in the ACC [39]. Therefore, we speculate that FMRP may alter the function of KAR, either directly or indirectly.

KARs may also play important roles in the insular cortex, similar to NMDAR or AMPAR. Indeed, KARs mediated currents are recorded in layer II/II pyramidal neurons of the adult mice insular cortex [19]. The KARs mediated currents require GluK1 and GluK2 receptor subunits because pharmacological blocking and/or gene deletions of both GluK1 and GluK2 receptors reduce the KARs mediated currents [19]. In the present study, we found that Fmr1 KO mice reduced KARs mediated currents induced by single and repetitive electric stimulations, suggesting that FMRP may be important in synaptic KARs mediated transmission in the insular cortex. Although Fmr1 KO mice did not alter total expressions of GluK1 and GluK2 receptors, lacking the *Fmr1* gene reduced these receptors in synaptosomes of the insular cortex. Combined with our biochemical finding, these results indicate that FMRP is crital for regulating the subsynaptic functions of GluK1 and GluK2 receptors in the insular cortex.

We further analyzed the possible molecular mechanism related to the abnormal KAR functions in Fmr1 KO mice using cultured cortical neurons. Both our electrophysiological and biochemical data indicate that knocking-out FMRP has no effect on the surface expression of GluKRs. Interestingly, we found that the kainate activity-induced endocytosis of GluK2 was blocked in the neurons with FMRP knock-out, indicating that the trafficking of KAR is affected. We are still unclear whether FMRP is involved in the regulation of KAR trafficking. Previous work in other labs has shown that the C-terminus of GluK2 may be modulated by protein kinase A (PKA) and/or protein kinase C (PKC)-mediated phosphorylation [40, 41]. The phosphorylation of GluK2 at Ser846 and Ser868 inhibits GluK2 from exiting the ER and trafficking to the cell surface [42]. Moreover, the phosphorylation of GluK2 at Ser846 accelerates KAR endocytosis from the plasma membrance and trafficking to the lateendosome. Importantly, Fmr1 KO mice showed enhanced activity of PKC [43–45]. Therefore, it is possible that the enhanced PKC activity in Fmr1 KO mice impairs the regulation of KAR trafficking. Further research is required to unveil the related signaling pathways.

## Abbreviations

ACC: anterior cingulate cortex
AMPAR: α-amino-3-hydroxyl-S-methylisoazole-4-propionate receptor
EPSCs: excitatory postsynaptic currents
FMRP: fragile X mental retardation protein
FXS: Fragile X syndrome
iGluRs: ionotropic glutamate receptors
KA: kainate
KAR: Kainate receptor
KO: knock-out
LTD: long-term depression
LTP: long-term potentiation
mGluR: metabotropic glutamate receptors
NMDAR: N-methyl-D-aspartate receptor
PFC: prefrontal cortex
PKA: protein kinase A
PKC: protein kinase C
WT: wild-type
